# Risk assessment and HbA1c measurement in Norwegian community pharmacies to identify people with undiagnosed type 2 diabetes – A feasibility study

**DOI:** 10.1371/journal.pone.0191316

**Published:** 2018-02-23

**Authors:** Aslaug Johanne Risøy, Reidun Lisbet Skeide Kjome, Sverre Sandberg, Una Ørvim Sølvik

**Affiliations:** 1 Department of Global Health and Primary Care, Faculty of Medicine, University of Bergen, Bergen, Norway; 2 Centre for pharmacy, Faculty of Medicine, University of Bergen, Bergen, Norway; 3 Norwegian Quality Improvement of Laboratory Examinations (Noklus), Haraldsplass Deaconess Hospital, Bergen, Norway; 4 Laboratory of Clinical Biochemistry, Haukeland University Hospital, Bergen, Norway; Florida International University Herbert Wertheim College of Medicine, UNITED STATES

## Abstract

**Objectives:**

Determine the feasibility of using a diabetes risk assessment tool followed by HbA1c-measurement in a community-pharmacy setting in Norway.

**Methods:**

In this longitudinal study two pharmacists in each of three community pharmacies were trained to perform risk assessments, HbA1c-measurements and counselling. Pharmacy customers who were > 18 years old and could understand and speak Norwegian or English were recruited in the pharmacies during a two-months-period. Information about the service was presented in local newspapers, social media, leaflets and posters at the pharmacy. Customers wishing to participate contacted the pharmacy staff. Participants completed a validated diabetes risk test and a background questionnaire including a validated instrument for self-rated health. A HbA1c measurement was performed for individuals with a moderate to high risk of developing diabetes. If HbA1c ≥ 6.5% they were recommended to visit their general practitioner for follow-up. The pharmacies performed internal and external quality control of the HbA1c instrument.

**Results:**

Of the 211 included participants 97 (46%) were > 50 years old. HbA1c was measured for the 47 participants (22%) with high risk. Thirty-two (15%) had HbA1c values < 5.7%, twelve (5.4%) had values between 5.7%—6.4%, and three (1.4%) had an HbA1c ≥ 6.5%. Two participants with HbA1 ≥ 6.5% were diagnosed with diabetes by their general practitioner. The third was lost to follow-up. Results from internal and external quality control for HbA1c were within set limits.

**Conclusion:**

The pharmacists were able to perform the risk assessment and measurement of HbA1c, and pharmacy customers were willing to participate. The HbA1c measurements fulfilled the requirements for analytical quality. Thus, it is feasible to implement this service in community pharmacies in Norway. In a large-scale study the inclusion criteria should be increased to 45 years in accordance with the population the risk test has been validated for.

## Introduction

It is estimated that approximatly 100 000–200 000 Norwegians have undiagnosed type 2 diabetes (T2D) [1]. The disease can develop slowly and the symptoms can be diffuse, therefore people with undiagnosed T2D have a tendency to underestimate their risk of this disease [2]. It can take 4–6 years before the disease is identified [3]. Nevertheless, with early identification and good treatment, it is possible to prevent or to delay long-term complications [4], such as diabetic retinopathy, neuropathy and nephropathy and cardiovascular disease [5].

For early identification of people with T2D and those at risk of T2D, screening can be performed [6]. Although there is some discussion about the utility of screening for T2D [7], a systematic review concluded that this is cost-effective and could be cost-saving [8]. On the other hand, exposing a large segment of the population to screening for T2D may lead to fear—two systematic reviews found that screening could increase the short-term anxiety [9, 10]. However, participants reported no long-term negative psychological impact [9, 10].

Both the World Health Organization [11] and the American Diabetes Association [12] open up for targeted screening for those with a risk of T2D. In Norway it is recommended to identify people with a high risk of developing T2D (case-finding [13]) [13, 14]. The Norwegian National Guidelines for Diabetes [14] recommend to use the validated Finnish Diabetes Risk Score (FINDRISC) as the first step [15]. If the FINDRISC score is high, a HbA1c (hemoglobin A(1c))-measurement should be performed. The HbA1c-test has several advantages compared with other available tests [16, 17] and is the recommended test for diagnosing T2D in Norway [14]. Because non-European ethnic groups have a different risk profile than ethnic Europeans, the Norwegian Diabetes Association recommends the diabetes risk test developed by Diabetes UK [18, 19] for this group.

Community pharmacy has been described as a low threshold service, due to long opening hours, and easily accessible health personnel that can be reached without an appointment [20]. A Swedish study found that over 70% of pharmacy customers think that pharmacies can motivate people to improve their health [21]. Furthermore, in a systematic review it was found that pharmacies are a feasible location to perform screening for T2D [22]. However, this has not been explored in Norway until now, and few studies have explored the use of HbA1c for screening in a pharmacy setting [23–25]. Before implementation of a complex intervention in a large-scale study, it is recommended to perform a feasibility study to optimize the protocol [26]. Thus, the objective with this study was to test the feasibility of using a diabetes risk assessment tool followed by HbA1-measurement in a Norwegian community-pharmacy setting.

## Methods

### Pharmacy recruitment

The study was performed in cooperation with Apotek 1, one of the three main pharmacy chains in Norway. Three pharmacies were chosen by the pharmacy chain’s pharmaceutical advisors. The participating pharmacies represented both urban and more rural settings, and included an area with a high prevalence of people with non-European background. Two pharmacists at each pharmacy were responsible for performing the diabetes risk assessments.

### Pharmacist training program and quality control of HbA1c measurements

Before recruitment of participants, the study pharmacists received detailed procedures for the risk assessment service developed by the authors. In addition, the pharmacists completed a training program that included two online courses developed by The Norwegian Quality Improvement of Laboratory Examinations (Noklus), covering capillary blood sampling and quality assurance, and a one-day course of face-to-face training. The course day included a detailed presentation of the procedures for the risk assessment service, risk communication training, practical demonstration of how to measure HbA1c with DCA Vantage Analyser (Siemens Healthcare Diagnostics, Erlangen, Germany) and how to perform analytical quality control of the measurements. The HbA1c instrument DCA Vantage is traceable to the International Federation of Clinical Chemistry (IFCC) reference measurement method [27]. The pharmacies were enrolled in Noklus’ EQA (external quality assurance) program for HbA1c. Before the inclusion of participants, a Noklus laboratory advisor visited each pharmacy to ensure that the pharmacists performed the capillary blood sampling and HbA1c measurement correctly. Internal quality controls for HbA1c were performed every week, and external quality controls were analysed twice during the study. The internal quality controls were evaluated against the limits given by the manufacturer of the instrument (HbA1c 4.2%—6.4% for the low control and 8.3%—12.5% for the high control). In the EQA survey for HbA1c the participants are evaluated for trueness and precision. The trueness is evaluated as “Very good” when deviation of results from the target interval (target value ± 0.1) is within ± 2% of the target interval and as “Acceptable” (target value ± 0.1) is within ± 7%. Precision is given by the difference between the duplicate measurement of the controls and is considered “Very good” when the difference is equal to or less than 0.2 and as “Acceptable” between 0.3–0.4 (limits given by Noklus). After the course day and the visit by a Noklus laboratory advisor, the project leader visited the pharmacies to make sure they were ready to start the inclusion of participants.

### Follow up during inclusion

After the first week of inclusion, a skype meeting was arranged with the project leader and the six study pharmacists to discuss the service, if something should be changed, and to allow study pharmacists to share experiences and helpful tips. During the first month of recruitment the project leader called the study pharmacists once a week for an update on recruitment progress and to answer any inquires.

### Participant recruitment

Participant recruitment was carried out from 22 of February to 22 of April 2016. Information about the service were covered in two regional newspapers, and then spread in social media (Facebook). The inclusion criteria were age over 18 years, and being able to understand, read and write Norwegian or English. Exclusion criteria were pregnancy, blood diseases that may influence measurement of HbA1c, or any diagnosis of diabetes. Leaflets and posters with information about the service were available at the participating pharmacies, and at other nearby pharmacies from the same pharmacy chain. Customers wishing to participate contacted the pharmacy staff and received oral and written information about the study. They signed an informed consent before entering the study. The participants were given identification numbers, and names were not disclosed to the project leader. Due to low recruitment after three weeks, the ethics committee approved that the pharmacists could give leaflets to customers, and inform them directly about the service. The service was free for the participants. The pharmacies received 100 NOK for each participant they recruited payed by the project funding.

### Background questionnaire including self-rated health

The participants filled in a questionnaire with information about their level of education, time since last visit to their GP (general practitioner), the reason why they wanted to be tested for diabetes and where they had heard about the service. The questionnaire also contained a standardised and validated instrument for measurement of self-rated health that day, on a continuous scale from 0 (worst imaginable health) to 100 (best imaginable health) [28].

### The diabetes risk tests and measurement of HbA1c

Participants of native European descent filled in the diabetes risk test FINDRISC [15, 29], while participants of non-European descent filled in the Diabetes UK-test [18], to account for the higher background risk in these ethnic groups [18]. Both tests are recommended in the Norwegian National Guidelines for Diabetes [14] and they included questions about age group, level of physical activity, weight ect. European participants with a high and very high risk of developing diabetes within the next ten years [15] and non-European with a moderate and high risk [18] were offered a HbA1c-measurement. If HbA1c was below 6.5%, participants were told that they did not have diabetes, and received general lifestyle advise as recommended in the national guidelines [14]. Participants with HbA1c 6.5% or higher were recommended to visit their GP for diagnosis and treatment.

### Follow-up questionnaires

European participants with low to medium risk and non-European participants with low to increased risk of developing diabetes within the next ten years, as well as participants with HbA1c was below 6.5%, received a follow-up questionnaire by e-mail or ordinary mail about two months after inclusion. Questions were:

- Had you considered that you could have diabetes before you took the risk test?

- Have you talked about diabetes with your doctor after you took the test?

- Are you more or less concerned about getting diabetes after taking this test?

- Is this a service you would recommend to others?

- What is positive/negative regarding diabetes risk testing at the pharmacies?

- Do you have suggestions for improvement of diabetes risk testing in pharmacies?

- Do you think pharmacies should offer this type of diabetes risk testing?

Furthermore, they were asked to fill in current self-rated health (as above). Participants with HbA1c with 6.5% or higher were asked the same questions by the pharmacist by phone. In addition, they were asked whether they had visited their GP as recommended, and if they there were diagnosed with diabetes.

### Statistics

All data from the questionnaires and diabetes risk assessments schemes were plotted into IBM SPSS Statistics (version 23 for Windows Software (Armonk, New York, USA)) for analysis. A histogram, Q-Q-plot and the Shapiro-Wilk-test was used to test if the data were normally distributed, a Shapiro-Wilk test p < 0.05 rejected normal distribution. Since self-rated health did not have a normal distribution, the Wilcoxon signed rank test was used for testing any significant difference between the self-rated health before the risk assessment test, compared with self-rated health two months after the test. The significance level was set at p < 0.05.

### Ethical approval

The study was approved by the Regional Committees for Medical and Health Research Ethics (REK/2015/2322).

## Results

Pharmacy A recruited 152 participants, pharmacy B recruited 36 participants and pharmacy C recruited 31 participants, which gives a total of 219 participants. Seven participants were excluded due to missing data (the risk assessment was not retained at the pharmacy) and one because he already had been diagnosed with diabetes, giving a final number of 211 participants. The background questionnaire, consisting of variables such as highest completed education, why the participant took the test, and where they had heard about the service, in addition to self-rated health, was completed by 198 (94%) of the participants. One hundred and eighty-seven (89%) filled out FINDRISC, while 24 (11%) filled out Diabetes UK. Both tests were available in a Norwegian and an English version; 189 (94%) participants filled in the form in Norwegian, while 13 (6%) used the English version. All participants completed the diabetes risk test. Table 1 shows the distribution of gender, age groups and levels of education by ethnicity among the participants.

**Table 1:** General characteristics of the participants.

	Diabetes risk test, n (%)		Total n (%) n = 211
	FINDRISC n = 187	Diabetes UK n = 24	
Gender			
Women	118 (63)	10 (42)	128 (61)
Men	69 (37)	14 (58)	83 (39)
Age groups (years)*			
< 45	80 (43)		
45–54	36 (19)		
55–64	28 (15)		
>64	43 (23)		
< 49		17 (71)	
50–59		6 (25)	
60–69		1 (4)	
> 70		0 (0)	
Level of education			
Primary school	14 (8)	3 (13)	17 (8)
High School	72 (39)	9 (38)	81 (38)
Bachelor	50 (27)	7 (28)	57 (27)
Master or higher	26 (14)	5 (21)	31 (15)
Other	4 (1)	0 (0)	4 (2)
Missing**	21 (11)	0	21 (10)

* The exact age is not available and age groups were slightly different in the risk assessment forms for FINDRISC [15] and for Diabetes UK [18], and are therefore shown separately.

**One pharmacy mainly filled in the background questionnaire for participants with high or very high risk, therefore the level of education is missing for 20 of the 23 participants with a low or medium risk recruited at this pharmacy.

### Risk of developing diabetes

Table 2 shows the distribution of risk of developing diabetes among the participants, self-rated health and HbA1c at inclusion. The median score for self-rated general health at inclusion was 70 (range 20–100, n = 183). Seventeen (9%) of the 198 participants who answered the background questionnaire did not complete the question regarding self-rated health. Thirty-two (15%) participants had HbA1c values < 5.7% (normal), twelve (5.7%) had values between 5.7%—6.4% (pre-diabetes), while three (1.4%) had an HbA1c ≥ 6.5% (cut-off for diabetes) [30]. The three HbA1c values ≥ 6.5% were 6.5%, 8.3% and 10.5%, and the corresponding score at the diabetes risk test were 19, 19 (both FINDRISC) and 18 (UK Diabetes).

**Table 2:** Results from the diabetes risk tests by ethnicity (FINDRISC [15] and diabetes UK [18]) and the corresponding statistical risk of developing diabetes type 2 within the next 10 years, self-rated health and HbA1c at inclusion.

Diabetes risk test	Total score	Statistical risk of developing diabetes type 2 within the next 10 years	n (%)	Self-rated health*	HbA1c, % (n)			
				Median** (range)	n	< 5.7	5.7–6.4	> 6.5
FINDRISC (n = 187)	< 7	Low: 1 of 100 develops the disease	53 (28)	73 (25–99)	44			
	7–11	Somewhat increased: 1 of 25 develops the disease	70 (37)	70 (25–99)	60			
	12–14	Medium: 1 of 6 develops the disease	31 (15)	70 (37–100)	28			
	15–20	High: 1 of 3 develops the disease	32 (17)	65 (20–100)	30	24	7	1
	> 20	Very high: 1 of 2 develops the disease	1 (1)	50	1		1	
Diabetes UK (n = 24)	0–6	Low: 1 of 20 develops the disease	4 (17)	81 (72–90)	3			
	7–15	Increased: 1 of 10 develops the disease	6 (25)	65 (45–80)	5			
	16–24	Moderate: 1 of 7 develops the disease	13 (54)	60 (50–95)	12	7	4	2
	25–47	High: 1 of 3 develops the disease	1 (4)			1		

*Self-rated health was missing for 28 participants

**Median, on a scale from 0–100.

### Follow up

The follow-up questionnaire was answered by 75 (35%) participants. Two participants with HbA1 ≥ 6.5% were diagnosed with diabetes by their GP, while the third was not reached for follow-up. There was no significant difference in general self-rated health before and two months after the risk test in any of the groups, regardless of risk score. Five (7%) of the 75 participants who were not advised to see their GP were more concerned about getting diabetes after they took the test, 37 (49%) were less concerned, 32 (43%) answered that there was no difference and one person (1%) did not know.

### The participants’ impressions of the service

In the background questionnaire 27 participants answered that they heard about the service at the pharmacy, 26 from family and acquaintances, 79 read about it in newspapers, 68 at websites or social media and six participants answered “other” (S1 Fig). In pharmacy A, 131 (87%) had read about the service in newspaper or social media (mostly Facebook), while for the two other pharmacies the numbers were nine (26%) and seven (28%). Eighty-seven participants responded that they wanted to take the test “Just to check”, 79 because someone in the family has diabetes, 73 because they have been concerned that they might have diabetes, 18 participants were encouraged by others and 21 participants answered “Other” (S2 Fig). More than one answer was permitted for both questions.

Most participants who responded to this question (n = 68, 90%), would recommend this service to others, while one participant (1%) would not, and seven (9%) did not know. There was an opportunity to fill in text in the follow-up questionnaire about the participants’ impression of the service. The responses could be divided into seven main positives aspects: Low threshold service (easy to access, fast, and easier than going to the GP); more people are diagnosed; you find out if you are in the risk zone for diabetes; you do not have to be concerned about having diabetes; you get more knowledge about diabetes; the test was of good quality; and that you are recommended to go to your GP if you have a high risk.

Most of the participants did not have any comments on possible disadvantages that the service could entail. The few comments that were given could be divided into five main categories: Uncertainty about whether the test was good enough; that not all participants received HbA1c measurements; the testing service could give rise to concern about getting diabetes; that it was non-committal/no follow-up; and that it could have been done faster. The participating pharmacists confirmed that the most common complaint among participants was that not all could have their HbA1c measured. However, improving the information prior to the risk test reduced the complaints.

The participants also made some comments that could be classified as prerequisites for the service. These could be divided into three main categories: That the staff have to be professional and discreet; high accessibility of the service and the option of booking an appointment; and that the room where the service was performed should be pleasant, private and uncluttered.

### The pharmacists´ impressions of the service

The pharmacists were satisfied with the training they received prior to the recruitment.

Despite this, some misunderstandings occurred. The pharmacists in one pharmacy assumed that only participants with a high risk of developing diabetes should fill in the background questionnaire at inclusion, and consequently there is a lack of information from 21 of the participants with a low, somewhat increased and medium risk (Table 1).

The pharmacists experienced that the DCA Vantage instrument for HbA1c measurement was easy to use, and found the visit and follow-up from the Noklus laboratory advisor useful. The pharmacists found that sending out the follow-up questionnaires was time consuming, and because not all the participants´ email addresses were readable, some participants did not receive the follow-up questionnaire.

### Analytical quality

In the EQA for HbA1c all three pharmacies scored “Very good” on trueness, two pharmacies scored “Very good” on precision, and the last pharmacy did not get any evaluation on precision because they did not send in two results so that the precision could be estimated. One of the pharmacies participated only in one of the two EQA assessments due to delays in the contract commitment with Noklus. The internal quality control scores were all within the limits given by the manufacture of the instrument.

## Discussion

This study shows that it is feasible to perform T2D case-finding using a diabetes risk assessment tool followed by HbA1-measurement in a Norwegian community-pharmacy setting. To our knowledge this is the first study in Norway where a screening protocol including both diabetes risk assessment test and measurement of HbA1c has been tested. Previous studies performed in other countries using a comparable protocol confirm the conclusion, however different risk assessment tools [24, 31, 32] were used. In addition, only one study used HbA1c-measurement [24], while glucose-measurement was used in the other studies [31, 32].

By using this protocol both people with moderate to very high risk of developing T2D (47 of 211 participants (22%)) and people with undiagnosed T2D (two of 211 (1%)) were identified. Our results also identified some points in the protocol that need optimization before a large-scale study is conducted.

A reason for offering this type of risk assessment in pharmacy, is that a large number of people visit the pharmacies. In Norway the pharmacies had about 50 million customers in 2015 [33]. The participating pharmacies in this study had an average of 300 customers a day in the inclusion period, which implies a total of potential 54 000 people during the study period. Still, many of the same customers visited the pharmacy multiple times during the inclusion period, thus the potential number of participants for this study was lower than the total number of customers. Surprisingly, only 13% of the participants had heard about this service when visiting the pharmacy, while 70% had read about it either in the newspapers/social media and 12% received information from family and friends. This could mean that that most of the participants were not typical pharmacy customers, but people who visited the pharmacy specifically to take the risk test. The fact that a lagre propotion of the participants were quite young, 80 under 45 years, and 17 under 49 years, supports this finding.

The pharmacists were satisfied with the training-program and the follow-up during the study. Furthermore, the results from EQA and internal quality controls show that the quality of the HbA1c-measurements was good. One study found that use of the HbA1c Point of Care (POC)-instrument was a barrier for the pharmacists, showing that training in the use of the DCA-instrument is an important part of the implementation of the service [24]. In our study online learning courses, a course day and follow-up by Noklus laboratory advisors, all contributed to ensure that the pharmacists felt competent to perform the HbA1c POC-testing at the pharmacy. However, the pharmacists did report that the procedure was time-consuming and that at least two pharmacists had to be at work at the same time to be able to perform the screening protocol undisturbed from the other customers and duties at the pharmacy. There were also periods were the project pharmacists were not available to perform the risk assessment.

There was a big difference in the number of participants recruited by the three pharmacies. Surprisingly, pharmacy A, which included the most participants (152), had a lower average number of customers per day (330), compared to pharmacies B and C, which included 36 and 31 participants, and had 457 and 359 customers per day. An explanation could be that the pharmacy with the lowest number of customers had more time to spend on this project and on recruitment. Moreover, the difference in recruitment could have been due to different levels of enthusiasm among the pharmacists. However an other possible reason could be that a regional newspaper wrote a article about pharmacy A offering the service and that this article was widely spread on social media (Facebook). The two other pharmacies did not receive similar media coverage.

We found no difference in the median score for self-rated health at inclusion and at follow-up, and 82% of the participants were not more concerned about getting diabetes after taking the risk test. Previous studies have also found that attending a screening programme does not have any long-lasting adverse psychological impact [34] and also that screening for T2D has limited impact on self-rated health [10, 35]. However, one study found that participants who screened positive had a poorer general health [34].

We used two different risk assessment tests, following national recommendations. In FINDRISC, it is recommended to measure HbA1c when the risk score is equal or over 15 and statistically one of three develops T2D within the next ten years [14]. While in the diabetes risk test from the Diabetes UK it is recommended to measure HbA1c when the score is equal or over 16 and statistically one of seven risk develops T2D within the next ten years [18]. We cannot see any reason why one chooses such different cut-off values, given that the total test-scores take into consideration the higher risk among non-Europeans. But we have chosen to follow the instructions of each test.

The use of validated measures for diabetes risk and self-rated health [36–38] was a strength of this study. Furthermore, the cut-off limits recommended for the diabetes risk tests was used, and the Norwegian National Guidelines for Diabetes was followed [14, 39, 40]. Additionally, the pharmacies were enrolled in Noklus’ EQA program for HbA1c ensuring that the measurement of HbA1c was quality assured. In Norway, the use of HbA1c POC-instruments for diagnostics purposes is allowed when the analytical performance specifications are fulfilled [14, 41]. External quality control of HbA1c- or blood glucose- measurements have not been implemented in comparable studies performed in pharmacies [24, 31, 32], even though this is recommended in guidelines [42, 43].

Eighty-seven percent of the participants were in the low-risk population and under the cut-off for HbA1c-measurement and only 1% were diagnosed with diabetes. One reason for this low risk profile could be that many of our participants were under 45 years old, while risk of T2D increases most after this age [1, 44]. The Diabetes UK is validated from 40 years old [18] and the FINDRISC test is validated for those who are 45 years or older [14]. Thus, 45 years old should have been the age limit for participation in the study. Including a higher share of participants with a non-European background would most likely have increased the percentage of participants with high risk of diabetes, since people with a non-European background have a higher prevalence of undiagnosed diabetes, also those living in Norway [1]. The share of participants with a non-European background varies from none to eight in similar studies [24, 32, 45]. In light of this, our 10% indicates that including a pharmacy in an area with a relativity high number of customers with a non-European background as well as offering the risk test in English as well as Norwegian might have had some effect on inclusion. One third (8 of 24) of the participants with a non-European background took the risk assessment in English, and they had probably not been included if the risk assessment was only available in Norwegian. We considered offering the risk assessment as well as the flyers and posters in languages such as Arabic and Somali in addition to English and Norwegian to increase the share of participants with a non-European background. However, we could not ensure that the pharmacist could convey relevant risk information to participants who could not communicate in English or Norwegian. Both different languages and other cultural differences have been found to be barriers for communication between health care providers and immigrants [46]. In addition, to consult with health care providers for chronic diseases screening and illness prevention is unfamiliar for a lot of immigrants, who are accustomed to only seek health care when they are ill [47]. Another limitation of the study was that we only have the age group of the participants, and not the exact age. Thus, the mean age of the participants could not be calculated. Consequently, that should be changed before a larger study is conducted.

The service was positively received by the participants, and 90% of responders would recommend it to others. However, due to the low response rate of the follow-up questionnaire (35%), the participants who answered may not be representative for all participants, and the results of the follow-up questionnaire must therefore be interpreted with caution. Possible explanations for this low response rate are that not all participants received the follow-up questionnaire or a reminder because of high workload and because not all the participants´ e-mail addresses were readable. Lack of time for the pharmacists was also an issue in a previous study [24]. In a larger study the pharmacist could either write the e-mail address themselves, or check the handwriting while the participant is present to ensure that the e-mail address is correct, and a reminder must be sent shortly after the deadline.

## Conclusion

This study confirms that it is feasible to implement a diabetes risk assessment tool followed by HbA1c- measurement in community pharmacies in Norway. The service is feasible and acceptable to both pharmacies and responding participants. In a large-scale study the inclusion criteria should be increased to 45 years in accordance with the population the FINDRISC has been validated for, and to avoid inclusion of too many people at low risk of T2D. To ensure a successful implementation of the intervention in a large-scale study a customized training program must be provided, in addition to detailed instructions and follow-up by the project leader. Furthermore, the pharmacy must provide enough time for both testing and follow-up of the participants. The pharmacies must perform internal quality control and participate in external quality control program to ensure analytical quality of the HbA1c instrument.

## Supporting information

S1 Fig Where did you hear about this service (that you can test your diabetes risk at the pharmacy)? (PDF)

S2 Fig Why do you want to take a diabetes risk test? (PDF)
