# Supplementary material for: Risk assessment and HbA1c measurement in Norwegian community pharmacies to identify people with undiagnosed type 2 diabetes – A feasibility study
Source: PLoS One. 2018 Feb 23;13(2):e0191316. doi: 10.1371/journal.pone.0191316 (PMC5825028; doi:10.1371/journal.pone.0191316)
Supplement: S1 Fig — (PDF) [file pone.0191316.s001.pdf]

## Appendix 1.

4. Where did you hear about this service (that you can test your diabetes risk at the pharmacy)?

(You can tick off several alternatives)

☐ A. Here at the pharmacy

☐ B. From family or acquaintances

☐ C. Read about it in the newspaper

☐ D. Through websites or social media

☐ E. Other, please specify ________________________

Here are the results from the category “other”:

| English | Number |
|---|---|
| Apotek 1 Landås<br>(another pharmacy in the same pharmacy chain) | 1 |
| Facebook | 1 |
| From a friend | 1 |
| Radio | 2 |
