# Supplementary material for: Risk assessment and HbA1c measurement in Norwegian community pharmacies to identify people with undiagnosed type 2 diabetes – A feasibility study
Source: PLoS One. 2018 Feb 23;13(2):e0191316. doi: 10.1371/journal.pone.0191316 (PMC5825028; doi:10.1371/journal.pone.0191316)
Supplement: S2 Fig — (PDF) [file pone.0191316.s002.pdf]

S2 Appendix 2

Question 5. On Background Questionnaire:

Why do you want to take a diabetes risk test?

(You can tick off several alternatives)

☐ A. Just to check

☐ B. Because I have family who has diabetes

☐ C. I have been worried I might have diabetes

☐ D. I was encouraged by others

☐ E. Other, please specify-

Here are the 21 answer from the “other” category

Blood sugar drops suddenly, I do need food
I think I have an unhealthy lifestyle
I had gestational diabetes and I am at risk
I have been at the borderline at a visit to my GP
I have ME / CFS and has many different symptoms. It is not easy for me to distinguish what is what, if I should have diabetes.
I have been very tired over time
I recognized some of the symptoms
I read about it and thougt that the symptoms fit
I read an article about the study in the newspaper, and recognized some of the symptoms of diabetes.
Much dizzy, reacts to sugar.
Because of tiredness
I do not feel well after eating sugar

Very thirsty, I go to the toilet a lot when I have drunk so much. I wake up at night and must I have something sweet - fast
I heard about the project
I am eating a lot of chocolate and rice
My mom is at risk
Elevated blood sugar during testing
PCOS, thus I have been told that I am predisposed
To chech if I am predisposed to get it
I think I eat a lot of sugar, 0.5 kg a week. Much carbohydrate.
I was in Spain with a doctor 3 years ago, diabetes was detected
